# Smart Bioinks for the Printing of Human Tissue Models

**DOI:** 10.3390/biom12010141

**Published:** 2022-01-15

**Authors:** Zeina Maan, Nadia Z. Masri, Stephanie M. Willerth

**Affiliations:** 1 Department of Mechanical Engineering, University of Ottawa, Ottawa, ON K1S 5J6, Canada; zmaan070@uottawa.ca; 2 Division of Medical Sciences, University of Victoria, Victoria, BC V8W 2Y2, Canada; nadiam@uvic.ca; 3 Department of Mechanical Engineering, University of Victoria, Victoria, BC V8W 2Y2, Canada; 4 Centre for Advanced Materials and Technology, University of Victoria, Victoria, BC V8W 2Y2, Canada; 5 School of Biomedical Engineering, University of British Columbia, Victoria, BC V6T 1Z4, Canada

**Keywords:** 3D bioprinting, biomaterials, small molecules, controlled release, drug delivery, stem cells

## Abstract

3D bioprinting has tremendous potential to revolutionize the field of regenerative medicine by automating the process of tissue engineering. A significant number of new and advanced bioprinting technologies have been developed in recent years, enabling the generation of increasingly accurate models of human tissues both in the healthy and diseased state. Accordingly, this technology has generated a demand for smart bioinks that can enable the rapid and efficient generation of human bioprinted tissues that accurately recapitulate the properties of the same tissue found in vivo. Here, we define smart bioinks as those that provide controlled release of factors in response to stimuli or combine multiple materials to yield novel properties for the bioprinting of human tissues. This perspective piece reviews the existing literature and examines the potential for the incorporation of micro and nanotechnologies into bioinks to enhance their properties. It also discusses avenues for future work in this cutting-edge field.

## 1. Introduction

3D printing is a manufacturing technique based on a set of processes that creates physical objects by adding layers of material corresponding to successive sections of a computer-aided design (CAD) model [1]. Engineers and designers have been developing innovative applications using 3D printing as it is a rapid prototyping, mass-customizable process that enables the creation of complex geometries that are impossible to achieve by other manufacturing methods [2]. In recent years, 3D printing has become financially feasible at the small enterprise level, giving this type of process a chance to move from heavy industries to office environments. This progress allowed additive manufacturing to have new applications in the medical sector. Thus, advanced 3D printing no longer only involved making three-dimensional objects using plastics and metal alloys, but also human cells, leading to a breakthrough technology known as 3D bioprinting. 3D bioprinting has attracted significant attention in recent years due to its potential to enable the rapid production of tissue-engineered constructs [3].

3D bioprinting attempts to reproduce the three-dimensional organization of cells, replicating what the human body naturally does. This process can fabricate custom tissues or organs using patient-derived cells, thereby minimizing the risk of rejection after transplantation [4]. The final product usually consists of an assembly of specific cells based on a predefined digital design produced in a layer-by-layer fashion [5]. 3D bioprinting offers unprecedented adaptability in positioning cells and creating environments with precise control over their compositions, spatial distributions and architectural precision, allowing for a detailed reconstruction of printed tissues and organs [6]. This technology is already used for the production of several types of tissues, and there are examples in the literature for generating multi-layered skin [7], bones [8], vascular grafts [9], neural tissues [10], heart tissue [11] and cartilage structures [12]. Researchers also have employed 3D bioprinting to produce organs, such as mouse ovaries. For example, sterile mice implanted with artificial ovaries were able to ovulate, give birth and feed healthy baby mice in the normal way, demonstrating the potential to generate organs [13]. 

Compared to non-biological 3D printing, 3D bioprinting requires additional levels of consideration, such as the choice of cell-adequate nutrient medium, cell type and growth and differentiation factors [14]. Bioprinting requires a three-stage process to make a bioprinted structure. The first stage consists of (i) selecting materials, (ii) formulating a printable ink and (iii) generating sufficient quantities of bioink for printing. A bioink is a mixture of materials and biological molecules or cells to be used for bioprinting [15]. Most bioinks are hydrogels, highly hydrated polymeric networks used to homogenously encapsulate cells by mimicking the natural extracellular matrix found in vivo. Hydrogels must meet certain characteristics to ensure they can support cell survival and function. These characteristics include: 

1–Rheological: Shear rate, shear stress, viscosity, and critical shear point of the bioink are the most significant rheologic parameters to assess if the bioink exhibits shear-thinning behaviour for improved print quality without impacting the bioink’s internal bonding structure [16]. In other words, the bioink’s rheological behaviour describes and informs the biofabrication process, resulting in consistent printing outcomes [17].

2–Mechanical: The potential to regulate the mechanical characteristics of a bioink, such as elastic modulus, shear elastic modulus, Young’s modulus, or degradation rate [18]. Controlling the stiffness of the hydrogel bioink enables extrusion-based biofabrication using a soft gel, which may then be strengthened afterwards by secondary crosslinking to enhance stability, as stated above [19].

3–Biocompatibilitys: Biocompatibility is described as a substance’s ability to retain high cell viability, promote cell growth and proliferation, and preserve the cell population’s healthy, distinctive phenotype after exposure to a material [20].

Making a hydrogel-based bioink requires that the desired cells are obtained in sufficient quantities and then added to the ink. The bioprinted structure must then be cultured in a medium enriched with nutrients designed to promote the appropriate cell growth and function. The selection of materials has a great impact on the biocompatibility, cellular viability, and mechanical behaviour of a bioprinted structure and thus care must be taken when determining the most suitable bioink for a given tissue engineering application. Most bioinks rely on crosslinking to turn the liquid bioink into a gel-like substance containing cells. Extrusion-based bioprinting often builds the desired structure through the addition of layers of cell-laden bioinks crosslinked to achieve the expected structures and their associated mechanical properties. Finally, these bioprinted constructs are cultured in media often followed by cellular and mechanical tests to characterize their constructs [21]. Accordingly, formulating appropriate bioinks with the complete required properties for the building of engineered functional tissues and organs is one of the most significant challenges of 3D bioprinting for tissue engineering. As a result, certain bioinks must be functionalized or modified in order to generate the most suitable bioarchetypes [22]. 

The use of functionalized bioinks in tissue engineering has resulted in a number of achievements for the healthcare industry, among which examples in the literature are reviewed in Table 1. For instance, while autologous dermal–epidermal skin substitutes for people who have suffered a loss of skin tissue have been a subject of research for almost 15 years, recently, 3D bioprinting has shown potential for wound repair and regeneration with bionic skin customized shape as well as cells and other materials distributed precisely [23]. Namely, Lee et al. used Kupffer cells (KCs) and human foreskin fibroblasts (FBs) encapsulated in collagen to mimic the dermal matrix of the skin. Histology and immunofluorescence showed that resultant tissue was morphologically and biologically representative of in vivo human skin tissue [24]. More recently, Albanna et al. developed a fibrin/collagen hydrogel carrier to directly transport allogeneic or autologous dermal Fbs and epidermal KCs to heal skin defects. This in situ bioprinting system exhibited accelerated wound closure in a mouse injury model [25].

The development of 3D bio-printing bone and cartilage tissues also opened potential doors to new treatments for patients with arthritis, bone fractures, dental infections, and craniofacial defects. For instance, Mei et al. used a photo-cross-linkable hydrogel and additives, including nanoparticles (NPs), functional cells, and drugs/cytokine to make a bio-ink for bioprinting bone and cartilage [31]. Such nanocomposite bioinks have more specified bioactivities and mechanical properties because they consist of polymer matrices embedded with nanoparticles (NPs), which can also work as a drug delivery system [32]. Different functional cells were introduced to the hydrogel, for example, osteoblasts, chondrocytes, bone marrow-derived mesenchymal stem/stromal (BMSCs)—which can differentiate into functional bone cells [33]—and articular cartilage-resident chondroprogenitor cells (ACPCs) for cartilage regeneration [34]. Cadena et al. also reviewed how 3D bioprinting has been used for central and peripheral nervous system modelling and therapeutic applications and summarized how different bioinks have been used for specific neural tissue applications [35]. Different research attempts use gelatin and alginate as a base component for the bioink, such as Monferrer et al., who used gelatin methacrylate and methacrylated alginate with neuroblastoma cells to quantify and localize the effects of physical–chemical communication signals between tumour cells and the surrounding biomaterial stiffness over time [30]. However, other studies used drug-loaded particles intending to provide an advanced tool for neural tissue engineering. Tao et al. dispersed drug-loaded poly(ethylene glycol)-poly(3-caprolactone) (MPEG-PCL) nanoparticles within a gelatin-methacryloyl (GelMA) hydrogel to form nerve conduits using a continuous digital light processing bioprinting technique to facilitate the nerve regeneration [36]. Sharma et al. generated bioprinted domes consisting of a novel fibrin-based bioink containing guggulsterone microspheres and hiPSC-derived neural progenitor cells (NPCs). This technology promotes cell differentiation into dopaminergic neurons when used to deliver small molecules like guggulsterone [37]. 

**Figure 1:**
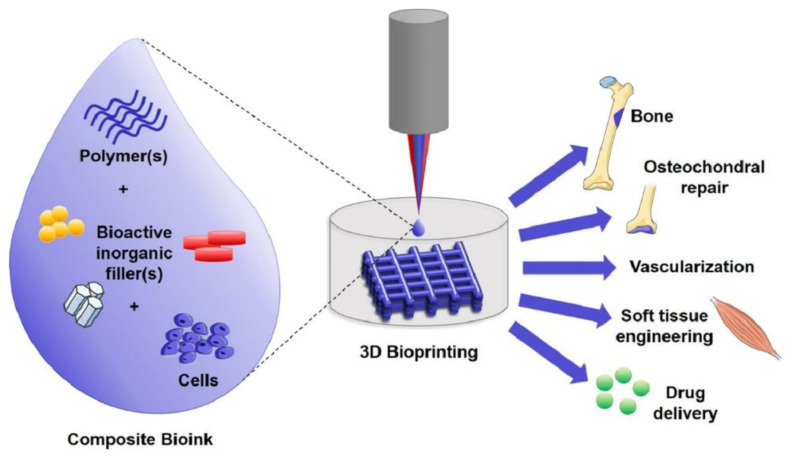
Schematic presentation of 3D bioprinting with composite bioinks. This image is reprinted under a Creative Commons CC BY 4.0 license from [41].

Overall, these aforementioned studies demonstrate the broad range of functionalized bioinks of different compositions used for potential applications for 3D bioprinting. A subset of functionalized bioinks would include composite bioinks as seen in Figure 1. These bioinks are created when certain particles are introduced into the cell-laden polymer matrix through mixing, allowing composites to support cell growth and avoid the introduction of shear forces during extrusion [38]. This perspective piece will discuss the concepts of “smart” bioinks, a type of composite bioinks capable of controlled release in response to stimuli that can be used as part of the 3D bioprinting process. These bioinks take advantage of drug-releasing particles to promote the desired behaviour of the cells being bioprinted following a detectable change in the physical or chemical structure of cells and their environment. Micro/nanotechnologies, in particular, have a vital role in enhanced medicine/drug formulations, targeted areas, and controlled drug release and delivery with great success [39,40]. Here, we review recent relevant literature in this area and highlight avenues for future expansion in the field. The advantages of such smart bioinks have the potential to transform the field of tissue engineering. 

## 2. Types of Smart Bioinks 

Various structures, including nanoparticles, nanofibers, microspheres, fillers and films, have been used for delivering drugs or therapeutic substances to tissues. These substances can promote cell growth, influence cell proliferation and differentiation, and control extracellular matrix (ECM) secretion [31]. For instance, statins [42], osteoprotegerin [43], v3 integrin antagonists [44], cathepsin K inhibitors [45], parathyroid hormone [46], transforming growth factor, and bone morphogenetic protein (BMP) [47] can stimulate bone growth. Angiogenic growth factors, vascular endothelial growth factors (VEGF), fibroblast growth factor, hepatocyte growth factor, and the platelet-derived growth factor have all been utilized to modulate blood vessel creation [31]. It is also critical to evaluate and optimize the chosen hydrogel’s biophysical and biochemical properties when 3D bioprinting functional tissues as they have a big impact on tissue behaviour and functionality [48]. The choice of bioink has a significant impact on the overall qualities of the printed constructs. For example, hydrogel-based bioinks often work not only as a structural substrate for printed tissue but also as a microenvironment for encapsulated cells, allowing them to direct their activities [49]. In this review, smart bioinks refer to drug-releasing particles found in composite bioinks, with a specific focus on bioinks with controlled drug release. Local delivery of biomolecules, including drugs and growth factors, have been demonstrated in different studies, which we have classified below according to the size and type of release particles. These types of structures can be incorporated into traditional hydrogel-based bioinks to improve their functionality. 

### 2.1. Nanoparticles

**Figure 2:**
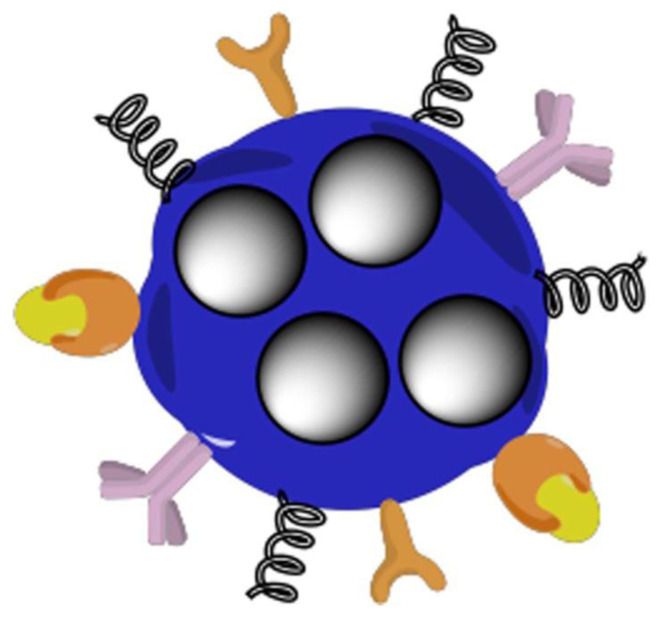
Representation of a smart multifunctional drug-loaded nanoparticle, decorated with various moieties for targeting, imaging and stealth properties. This image is reprinted under a Creative Commons CC BY 4.0 license from [53].

Nanotechnology describes research applications focused on the principles and properties existing at the nanometric scale, at the level of atoms and molecules [50]. The objective of nanotechnology is to produce objects or materials smaller than 100 nanometers [51]. These nanomaterials can be composed of nanoparticles, as seen in Figure 2, which are produced intentionally unlike very fine particles of natural origin. Individual molecules and interacting groups of molecules in relation to the bulk macroscopic properties of the material or device become important at the nanoscales because they have control over the fundamental molecular structure, allowing control over the macroscopic chemical and physical properties [52]. Heid and Boccaccini reviewed composite hydrogel bioinks with bioreactive inorganic fillers (BIFs) for 3D bioprinting. Isotropic and anisotropic silicates, such as bioactive glasses and nanoclays, as well as calcium–phosphates, such as hydroxyapatite (HAp), are BIFs that provide in situ crosslinking effects and additional functionality to the matrix. Due to their various chemical compositions, most BIFs can support cell adhesion and proliferation and some of them can also influence stem cell, progenitor cell, or cell line differentiation [41]. For instance, osteogenic cells are influenced by bioceramics, HAp, nanoclays, chondrogenic cells by bioactive glasses, angiogenic cells by some of the bioceramics, bioactive glasses and adipogenic cells by silica nanoparticles and neurogenic responses by carbon nanotubes [41].

Gungor-Ozkerim et al. provide an in-depth discussion of the different bioinks currently employed for bioprinting, including three composite bioinks incorporating bioactive molecules [54]. They created printable semi-synthetic extracellular matrix (sECM) hydrogels employing gold nanoparticles (AuNPs), thiol-modified biomacromonomers generated from hyaluronic acid (HA), and gelatin as dynamically cross-linkable components. AuNP-sECMs, a hydrogel having both intra-gel and inter-gel covalent interactions, developed from this mixture. AuNP-sECMs encapsulated NIH 3T3 fibroblasts and were used to 3D bioprint tubular tissue constructs. The cellular rings grew visibly opaque as cells proliferated and secreted the ECM over the four-week culture period. Similar specific staining was seen in positive control tissues. However, the presence of collagen in the tissue constructs indicates that the bioprinted cells reconfigured their environment [55]. Similarly, Zeng et al. used mussel-mimetic adhesion to incorporate Gold nanorods (AuNRs) coated with polydopamine (AuNR-PDA) into a thermosensitive injectable hydrogel composed of β glycerophosphate-bound chitosan (CGP) and dopamine-modified alginate (Alg-DA). Because of the strong contacts between polydopamine (PDA) and polymers, AuNR-PDA was able to be immobilized firmly and uniformly in the CGP/Alg-DA/AuNR composite hydrogel, preventing overheating or leakage. Under several trials of photothermal therapy, the in vitro cytotoxicity test of the composite hydrogel revealed high biocompatibility to normal cells, mouse fibroblasts, but clear suppression of human hepatocellular carcinoma (HepG2) cells. Furthermore, the in vivo antitumor test revealed that the CGP/Alg-DA/AuNR composite hydrogel inhibited tumour development significantly under several photothermal therapies (PTT). As a result, the injectable CGP/Alg-DA/AuNR hydrogel could be a strong choice for lengthy photothermal tumour treatment [56].

Likewise, Gao et al. co-printed nanoparticles of either bioactive glass (BG), hydroxyapatite (HA), or both BG and HA with bone marrow-derived human mesenchymal stem cells (hMSCs) suspended in a poly(ethylene glycol) dimethacrylate (PEGDMA) scaffold that was simultaneously polymerized to stimulate osteogenesis. In comparison to the other groups, hMSCs’ interaction with HA showed the highest cell survival and compressive modulus after 21 days in culture. The PEG-HA scaffold produced the most collagen, had the most alkaline phosphatase activity, and expressed the most collagen deposition, according to biochemical analyses. As a result, HA nanoparticles were more effective than BG in promoting hMSC osteogenesis in bioprinted tissues [57]. Catros et al. used LaBP while assembling nano-hydroxyapatite (nHA) and human osteoprogenitors (HOPs) in a culture medium in two and three dimensions. During LaBP, nHAp’s physicochemical qualities were retained, and cell proliferation was detected after 15 days [58]. On the other hand, Castro et al. used two biologically inspired nanomaterials: (i) osteoconductive nanocrystalline hydroxyapatite (nHA), being the primary inorganic component of bone; and (ii) core-shell poly(lactic-co-glycolic) acid (PLGA) nanospheres. The two components were bioprinted into a porous and highly interconnected osteochondral scaffold with hierarchical nano-to-microstructure and spatiotemporal bioactive factor gradients after being mixed with chondrogenic transforming growth factor (TGF-1) for sustained release. Human bone marrow-derived MSC adhesion, proliferation, and osteochondral differentiation were all considerably improved in the biomimetic graded 3D bioprinted osteochondral construct in vitro, according to the findings [59].

Ye et al. developed a drug-release delivery system to inhibit the growth and recurrence of osteosarcoma by incorporating hydrophobically modified silica nanoparticles (m-SiO_2_)/poly(ε-caprolactone) (PCL) porous scaffolds into ruthenium-loaded PEGylated liposomes (RL) to obtain a Ruthenium-loaded PEGylated liposome scaffold (RLS) composite as a result. The results show that the scaffold had a porous structure that is rather consistent and has good mechanical properties. Over the course of 48 h, the medication was released from RLS in a relatively consistent manner. RLS caused a mitochondrial malfunction, which lead to apoptosis in human osteosarcoma cancer cells (MG-63). All of the findings suggested that RLS could be a promising therapy option for osteosarcoma [60]. Similarly, Shu et al. incorporated a poly(N-isopropylacrylamide)-co-(acrylic acid) (pNIPAm-co-AAc) microgel with CuS nanoparticles (CuSNPs) to destroy cancer cells using near-infrared techniques (NIR). The solution of hybrid microgels revealed non-cytotoxic hybrid microgels that may be employed to kill HeLa cells using NIR excitation. When HeLa cells were treated with 400 g/mL hybrid microgels and subjected to 808 nm laser light with a power density of 2 W/cm^2^ for 10 min, nearly 90% of the cells were destroyed. While these materials have the potential for photothermal therapy, they can also be combined with a hydrogel matrix to stimulate the release of small molecule drugs when exposed to NIR wavelengths [61]. Such technologies could also be incorporated in bioinks as well. 

Additionally, Lee et al. study the printability of a polymer-based bioink based on dynamic covalent linkages between nanoparticles and polymers. For that, amine-presenting silica nanoparticles (SiNPs) were added to a polymeric ink containing oxidized alginate (OxA) and the mix was used to bioprint gels containing chondrocytes. The formation of reversible imine bonds between amines on the nanoparticles and aldehydes of OxA provoked considerably enhanced rheological properties resulting in the generation of porous constructs and an ear structure with overhangs and high structural fidelity. The improvement was mostly due to electrostatic interactions between cationic SiNPs and anionic polysaccharides and was significantly impacted by the size and concentration of the nanoparticles as well as the length of polymer chains [62]. This study indicates that these interactions should be considered when bioprinting cartilage. Moreover, Chimene et al. developed a bioactive nanoengineered ionic–covalent entanglement (NICE) bioink [63]. The NICE bioinks use nanosilicates to reinforce an ionic–covalent entanglement hydrogel made from methacryloyl (GelMA) and kappa–carrageenan (κCA), creating a dually reinforced hydrogel network to bioprint a preosteoblast-encapsulated scaffold as a way to engineer bone tissue. The interactions between GelMA and κCA allowed the NICE bioink to behave like a solid at low shear pressures, improving shear thinning properties. Due to the ionic–covalent entanglement and nanosilicate reinforcement, the printed material demonstrated high cell survival as well as a significant increase in mechanical strength of hydrogel structure. The NICE bioink can be used to print human-scale relevant 3D bioprinted structures such as cylindrical Y-shaped blood vessels and ears as well as in drug delivery and other applications of tissue engineering [63].

**Figure 3:**
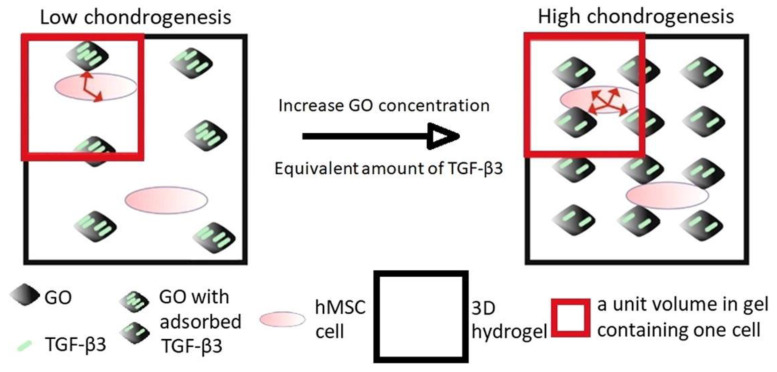
Demonstration of simultaneously incorporating both human mesenchymal stem cells (hMSCs) and GO (graphene oxide)-adsorbed growth factor TGFβ3 into a 3D scaffold, where GO-adsorbed TGFβ3 enhanced chondrogenic differentiation of hMSCs and cartilage-tissue synthesis This image is reprinted under a Creative Commons CC BY 4.0 license from [67].

Bakht et al. highlight recent achievements in the creation of functional nanocomposite hydrogels, focusing on their current and potential use as bioinks. This review discusses scientific progress in recent years, with a focus on bioinks with nanoparticles for regulated release of drugs and bioactive chemicals, as well as stimuli-responsive features with smart nanoparticles [64]. Among the reviewed articles, Fujioka-Kobayashi et al. applied an acryloyl group-bearing cholesterol-modified pullulan (CHPOA) nanohydrogel as a carrier of fibroblast growth factor 18 (FGF18) and/or bone morphogenetic protein 2 (BMP2) to critical-sized bone defects in mouse parietal bones to examine whether these growth factors (GFs) could cooperatively promote bone formation in vivo. The CHPOA/hydrogel acted as an excellent carrier for delivering two separate proteins, FGF18 and BMP2, and stimulated the healing of defects, according to the findings [65]. Moreover, Luo et al. integrated a bone-forming peptide-1 (BFP-1)-laden mesoporous silica nanoparticles (pep@MSNs) into an adhesion peptide comprising arginine-glycine-aspartic acid domain (RGD). Modified alginate hydrogel (RA) system (pep@MSNs-RA) was, consequently, created to encourage osteo-differentiation of hMSCs [66]. Furthermore, Zhou et al. employed graphene oxide (GO) nanoparticles to adsorb transforming growth factor β3 (TGF-β3), which were then integrated into a collagen hydrogel, as demonstrated in Figure 3. Chondrogenic differentiation was examined using hMSCs contained in the same gel. The findings show that GO flakes are an effective way to administer GFs in three-dimension to guide cells into the same scaffold and trigger tissue growth [67]. All these materials could potentially be translated for applications in bioprinting. 

Overall, these studies indicate how such smart bioinks employing nanoparticles can promote differentiation and cell survival. Drug nanoparticles possess increased solubility and hence better bioavailability due to their very small size and wide surface area [53] as well as the capacity to penetrate the blood–brain barrier (BBB), enter the pulmonary system, and be absorbed through the tight connections of skin endothelial cells [68]. Nanoparticles made from natural and synthetic polymers have been of considerable interest because they can be customized for targeted drug delivery, improved bioavailability, and controlled release of medication from a single dose; the system can also prevent endogenous enzymes from degrading the drug through adaptation [69].

### 2.2. Microparticles and Microspheres 

Microparticles, ranging in size from 1 to 1000 µm, can also serve as an effective tool for delivery, especially in the context of 3D bioprinting applications [70]. These particles can both be used as a tool for drug delivery or alone as an additive to modify the properties of the bioink. For example, Kim et al. synthesized dECM microparticles by decellularizing and freeze-milling a pig liver. This novel bioink, dECM powder-based bioink (dECM pBio-ink), was made by dissolving dECM micro-particles in a gelatin solution, and it outperformed the traditional bioink in terms of layer stacking for 3D bioprinting, while the conventional bioink could not keep its shape. Finally, in vitro studies with endothelial cells and primary mouse hepatocytes showed that the dECM pBio-ink had comparable cytocompatibility to the regular dECM bio-ink [71]. Neufurth et al. created a morphogenetically active bioink prepared of amorphous microparticles made of calcium ions (Ca^2+^) and polyphosphate (polyP), reinforced with poly–caprolactone (PCL). The resulting granular PCL/Ca-polyP-MP hybrid material was used to 3D bioprint tissue-like scaffolds with open pores for cell migration using a layered architecture. The printed composite scaffold had biomechanical properties similar to cortical and trabecular bone. Staining for cell viability, cell density, and scanning electron microscopy (SEM) analyses revealed that this scaffold could attract and foster the growth of human bone-related osteosarcoma (SaOS-2) cells. Based on the findings, it was determined that granular PCL/Ca-polyP-MP hybrid material is ideal for the production of bioprintable scaffolds with morphogenetic potential as well as biomechanical stability [72]. Finally, Sun et al. 3D-bioprinted a protein-releasing cell-laden Hydrogel-PCL composite scaffold to create an integrated live meniscus construct. Transforming growth factor β3 (TGFβ3) or connective tissue growth factor (CTGF) were carried in distinct sections of the hydrogel, encasing PLGA microparticles to produce anisotropic phenotypes to be bioprinted into the microchannels between PCL fibres from different syringes. In vitro and in vivo, the regenerated meniscus construct had cell morphologies and matrix deposition that were similar to the native anisotropic meniscus. Furthermore, the 3D-bioprinted meniscus gave long-term chondroprotection when transplanted into goat knees [73].

Microspheres are engineered materials defined as spherical or round-shaped microparticles [74]. They are often used during the bioprinting and post-printing processes because they cushion the cells, preventing shear stress from occurring, thus allowing different types of cells to grow in a more ideal 3D environment [75]. Chen et al. seeded PC12 and Schwann cells on a new hydrogel they created using Gelatin methacryloyl (GelMA) and Chitosan Microspheres (GC-MSs). The 3D multiscale composite scaffolds were bioprinted using microspheres and hydrogel as the modular bioink to test neurite outgrowth and Schwann cell proliferation as a way to engineer neural tissue. The findings show that a multiscale composite scaffold provided an adequate 3D microenvironment to improve neurite growth and that a 3D printed hydrogel network could offer a 3D macroenvironment that mimics the epineurium layer to proliferate Schwann cells and organize nerve cells, showing promise for neural tissue engineering applications [76]. Comparably, Nguyen et al. investigated the physical characteristics of gelatin methacrylate (GMA) microspheres on fibroblast-seeded hydrogels and their ability to release growth factors. With collagenase treatment, a lower GMA cross-linking density resulted in a more complete release of bone morphogenic protein 4 and basic cell growth factor, as well as a faster release rate. These findings show that GMA microspheres provide a more flexible platform for growth factor delivery by increasing relative binding capacity and allowing proteolytic degradation tunability, resulting in a more potent controlled release system [77]. Such microspheres could be incorporated into smart bioinks for similar applications. 

To make a highly viscous dispersion, Lohfeld et al. combined poly(lactic-co-glycolic acid) microspheres with agarose and hyaluronic acid hydrogel. To evaluate the effect of the fluid phase on sintering capabilities, the compounds were sintered with subcritical CO_2_. Despite the second phase, CO_2_ was found to be able to reach the microspheres and sinter them, resulting in a mechanically robust structure that can endure substantial forces. Microsphere technology allows controlled release of encapsulated factors to enhance tissue growth through the design of unit cells within a scaffold, and the printing process with multiple printing heads allows focal placement of various phases to account for different needs of tissue to create scaffolds that serve the growth of both cartilage and subchondral bone, as required for tissue-engineered joint replacements [78]. In parallel, Sharma et al. bioprinted dome-shaped constructs containing NPCs and incorporated guggulsterone drug-releasing microspheres in bioink as a method to induce cells to differentiate toward a dopaminergic neuronal fate and create a complex tissue model. This model resembles the microenvironment of the brain, which has a porous structure that allows nutrients and oxygen to circulate, permitting long-term cell culture in vitro. The findings of this study revealed that when NPCs are grown in a 3D environment, they react differently to guggulsterone. As a result, this microenvironment promotes the differentiation of NPCs into glial cells, along with tyrosine hydroxylase, a dopaminergic neuronal marker, in positive neurons. Furthermore, unloaded microspheres had the highest percentage of cells expressing an oligodendrocyte progenitor marker, implying that poly-ε-caprolactone microspheres favoured oligodendrocyte differentiation over neuron and astrocyte differentiation. Additionally, the presence of an oligodendrocyte progenitor marker in guggulsterone microsphere-containing tissues indicated that these tissues carried all three major neural subtypes: neurons, astrocytes, and oligodendrocytes [37]. This study demonstrates the successful use of a microsphere-laden bioink for engineering neural tissue similar to that found in the brain. 

To summarize, smart bioinks including microparticles and microspheres delivery systems are distinguished by certain characteristics such as (i) physical and chemical stability of the encapsulated active ingredient, which should be maintained throughout the process; (ii) simple, reproducible, and expandable manufacturing, ideally ending with optimal drug loading, maximum encapsulation, and maximum yield at the intended rate for an adequate time period; (iii) flowability and syringeabilitiy [79]. 

### 2.3. Microswimmers

Magnetic helical microswimmers, also known as artificial bacterial flagella (ABFs), are microscale devices/robots that use rotating, oscillating magnetic fields, or magnetic field gradients to swim in liquid. They transform rotational motion into translational motion to perform 3D navigation in diverse liquids under low-strength rotating magnetic fields. ABFs microswimmers have been extensively researched as carriers for selective drugs and cells. Control of individual groups of swimmers within a swarm is required for numerous biological applications such as drug delivery and release or small-scale surgery in vivo and in vitro [80]. Wang et al. fabricated GelMA microswimmers with user-defined geometry and added Fe_3_O_4_ magnetic nanoparticles to their surface to render them magnetically responsive, then, human skin fibroblast cells were cultivated on arrays of the GelMA microstructures. Unlike prior rigid helical microrobots, the soft helical microswimmers were able to corkscrew over the step-out frequency while maintaining relatively high advancing velocity, indicating an unparalleled self-adaptive capability. GelMA microswimmers were also discovered to be highly cell compatible. They are also entirely degradable by collagenases, promote cell adhesion and development, and are gradually degraded throughout a culture by cell-released enzymes. These non-cytotoxic biodegradable hydrogel microswimmers are great prospects for several applications in medicine and tissue engineering as they reduce the worry of collecting microrobots after drug-release procedures [81]. 

Chemical composition and surface functionalization are frequently required for nanosized drug delivery devices, and techniques for their functionalization for targeted drug delivery are being widely used [82]. While functionalization is possible with microswimmers, their unique shapes and multiple control mechanisms allow for extensive customization of the drug delivery process as compared to traditional micro/nanoparticles drug delivery systems [83]. Thus, this type of particle is an attractive candidate for use in functionalizing bioinks. 

### 2.4. Nano/Micro/Macrogels 

**Figure 4:**
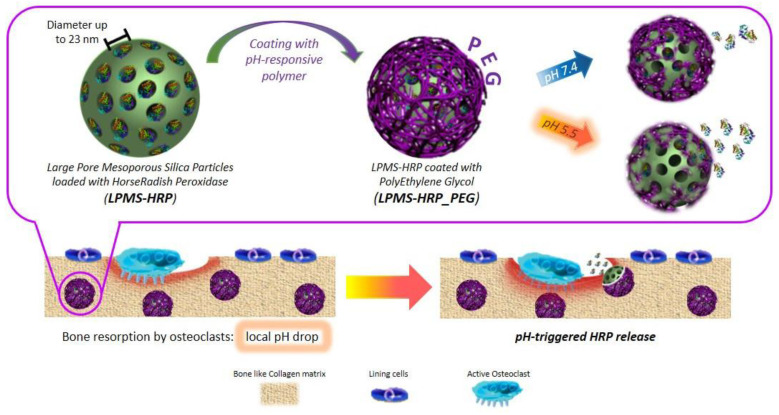
Schematic representation of the procedure of obtaining mPEG-silane on LPMS_HRP through PEGylation method. This image is reprinted under a Creative Commons CC BY 4.0 license from [85].

Hydrogels can regulate release performance by managing swelling or degradation thanks to their good compatibility and hydrophilicity [84]. Photo-polymerized hydrogels have, therefore, been employed for localized drug delivery depots for they can encapsulate cells, drugs, or nanoparticles and give physical support at the location of a printed tissue [31]. Banche-Niclot et al. developed large-pore mesoporous silicas (LPMSs) to transport large biomolecules and release them under a pH stimulation for use in bone regeneration [85]. The suggested pH-triggered approach intends to imitate the release of growth factors contained in the bone matrix because of bone resorption by osteoclasts (OCs) and the resulting pH drop in bone remodelling. To achieve this, large-pore mesoporous silicas were made using 1,3,5-trimethyl benzene (TMB) as a swelling agent, and the synthesis solution was hydrothermally treated to see how varied process temperatures and durations affected the final mesostructure. As summarized in Figure 4, LPMSs were coated with a pH-responsive polymer, poly(ethylene glycol) (PEG), to enable the transfer of the incorporated biomolecules in response to a pH decrease The results showed that in an acidic environment, PEG-coated carriers released horseradish peroxidase more quickly due to the protonation of poly(ethylene glycol) at low pH, which catalyzes the polymer hydrolysis reaction. The findings of this study, therefore, suggest that large-pore mesoporous silicas could be employed as carriers for large biomolecules and that poly(ethylene glycol) can be used as a pH-responsive coating [85]. Thus, this method of delivery might be adapted for 3D bioprinting. 

Highley et al. made their microgels out of norbornene-modified hyaluronic acid (NorHA), poly(ethylene glycol) diacrylate (PEGDA), and agarose, and cross-linked them with a photoinitiator and light (NorHA and PEGDA) or cooling (agarose) [86]. These three microgel types were chosen for their diversity, as they are made up of polymers that are both charged and uncharged to account for electrostatics, and they undergo different cross-linking mechanisms like radical chain-growth polymerization, thiol-ene photoinitiated cross-linking, and thermally induced physical cross-linking. NorHA microgels were created via thiol-ene cross-linking on a microfluidic device, as NorHA has previously been used to create hydrogels encapsulating cells such as MSCs for their tuned mechanics and controlled degradation [87,88]. Microgels were rinsed from the oil into buffer after cross-linking and had a uniform size distribution. Microgels were subsequently “jammed” by removing the aqueous medium between the particles via centrifugation over a filter or vacuum filtering, resulting in an extrudable ink containing clearly visible microgel components under microscopic examination. The general characteristics of jammed microgel inks are elastic response at low strains, yielding and flow as strain increases, shear-thinning behaviour, response to frequency changes, and the formation of stable filament structures after extrusion. Some similarity was shown for bioinks made from PEGDA and agarose microgels, though the magnitudes varied, suggesting that printability features are essentially a function of jamming and to some extent independent of the microgel composition [86].

Wang et al. present a 3D bioprinting technology to fabricate the shape memory hydrogels with internal structure (SMHs) by combining sodium alginate and pluronic F127 diacrylate macromer (F127DA) using a dual network layout. One layer is a reversible network generated by Ca^2+^ cross-linked alginate, while the other layer is a stable network formed by F127DA photo-crosslinking. After Ca^2+^ was removed from the Na2CO3 solution, SMHs showed a high recovery ratio, and the elastic modulus remained practically steady after the shape memory cycle. In vitro investigations revealed that the drug release rate is faster than with typical drug-loaded hydrogels. The vitality of 3T3 fibroblasts was not affected, indicating its high biocompatibility. As a result, it is predicted that SMHs have a bright future as medication transporters and tissue engineering scaffolds [89]. Finally, Wu et al. developed new scaffolds for the use of endothelial cell repair. 3D bioprinting was used to create a variety of biocompatible and biodegradable macroporous scaffolds with interconnected pores. To generate semi-solid viscous bioinks, various formulations of polylactic acid (PLA), polyethylene glycol (PEG), and pluronicF127 (F127) were prepared. As a model drug, either dimethyloxalylglycine (DMOG) or erythropoietin (EPO) was put into viscous biocompatible ink formulations. Investigations revealed that each scaffold had the optimum rheological and mechanical properties. Because DMOG is an HIF-1 inducer, its release into PBS solution was assessed indirectly using an HIF-1 bioassay, implying that the optimized bioprinted scaffolds demonstrated controlled release of both EPO and DMOG when employed separately. This research suggests that this technology can be used to create biodegradable composite scaffolds for possible clinical applications in endothelial cell healing in cardiovascular disease (CVD) or other illnesses that cause endothelial damage [90]. 

### 2.5. General Hydrogel-Colloid Composite Bioinks

A colloid is a dispersion of one or more substances suspended in a liquid, forming a system with two separate phases [91]. Colloids can be heterogeneous mixtures of nano- or microscopic particles of various shapes such as spheres, platelets, crystals, rods, and fibres [92]. Michel and Auzely-Velty give an overview of recent research on colloid composite bioinks for extrusion printing. They discuss how embedded colloids influence the rheology of bioinks and the mechanical characteristics of printed hydrogels, as well as the critical role of colloidal materials in regulating cell fate and adding novel functionalities to design sophisticated tissues [92]. Insights and methods for choosing the right colloidal components in the formation of composite bioinks are also presented. Finally, the review’s final section discusses remaining problems and promising future directions, including the potential of composite systems for 4D bioprinting and the formulation of bioinks including several colloidal species for printing multifunctional biomimetic tissues. The authors address a wide range of colloids, including hydroxyapatite, cellulose, silica, clay, graphene, and others, that interact favourably with cells and demonstrate distinct functionalities throughout the manuscript. Antioxidant properties, drug release, and electroactivity can all help guide and enhance synthetic tissue formation. For all of these reasons, it was concluded that colloidal materials serve a critical role in the creation of novel bioinks with improved printability that can produce complex tissue constructs [92].

Bhattacharyya et al. explained the parameter optimizations for semi-automated mixing of bioink components and 3D bioprinting with the twin-screw extruder head with alginate, alpha-tricalcium phosphate (α-TCP) micro/nanoparticles, and osteoblast cells [93]. The TSE-treated bioink samples outperformed the conventional ones in terms of bioprintability, mechanical properties, and biological properties. Even with continuous feeding and extrusion-based bioink printing, the micro/nanoparticles were uniformly dispersed in the bioink, and the live cell distribution in the printed structures was substantially better than conventional mixing. The control of consistent micro/nanomaterials and cell distribution throughout the directly mixed printed bioink was achieved with this novel extrusion head, with minimal cell damage. Due to their highly efficient variable screw pitch design, they also supplied increased batch uniformity in real-time mixing and bioink printing. Increased automation and reduced processing time resulted in higher repeatability than the traditional method of bioink component mixing and subsequent 3D bioprinting, showing a promising basis in tissue engineering applications through the controlled mixing of bioink components and subsequent 3D bioprinting without affecting the cells [93]. Alternatively, Wang et al. established a hybrid suspension combining Eudragit polyacrylate colloid as matrix material and Pluronic polyether hydrogel as a diffusion channel for drug/protein release. Because of its pseudoplastic and thixotropic rheological properties, this hybrid suspension can be 3D printed into complicated shapes and interior structures. The protein can be used in a hybrid solution in its natural form or as a nanoparticle encapsulated version. The protein release from the construct is a function of drying time, chitosan molecular weight (MW), and their own structural/diffusional features, according to the experiment. In addition, the theoretical derivation reveals that polyacrylate matrix tortuosity, chitosan erosion rate, and hydrogel diffusion coefficient all played a role in the drug release profile’s extended duration. Furthermore, cytotoxicity testing using cell culture indicated that the hybrid suspension construct is relatively biocompatible. Finally, as a protein delivery system, heterogeneous structures with the zoned design were created, demonstrating the possibility of the hybrid suspension technique to achieve pharmaceutical effectiveness or guild cell organization by the spatial and temporal release of macromolecular medicines [94]. 

Overall, composite bioinks hold significant potential in engineering tissues due to the ability to create multifunctional bioinks [95]. Numerous studies have combined synthetic and natural biomaterials to provide the resultant desirable composite physical and chemical qualities, such as strengthening the material or regulating shear-thinning properties [96]. The composite ink also showed significant cell concentration and vitality, as well as cellular differentiation with matrix deposition [97]. 

## 3. Conclusions

Bioprinting is an additive manufacturing technology that uses bioinks in combination with cells to produce living structures [98]. These bioinks are made up of cytocompatible hydrogel precursor formulations that gel in a compatible way with various bioprinting techniques. The printability of bioink depends on its properties before, during, and after gelation, which includes structural resolution, form fidelity, and cell survival [99]. These properties are regulated by the number of cells in the construct, their proliferation, migration, and contact with the material during tissue growth. A well-calibrated computational framework can forecast tissue regeneration while also optimizing bioprinting input parameters including the beginning material, initial cell loading, and construct design [99]. 

Recent advances in bioprinting provide a valuable tool to fabricate biomimetic constructs, which can be applied in different stages of drug release research. This paper presented many types of “smart” bioinks that can be used in 3D bioprinting. To encourage the desired behaviour of the cells being bioprinted, such bioinks use drug-releasing particles. After analyzing current research on this topic and identifying areas for further growth, it was concluded that “smart” bioinks and materials have the potential to revolutionize tissue engineering. For instance, in vitro experimental studies, though computational advances have resulted in more rationale computer-aided molecular design, still require gaining industry confidence and improving in vitro-in vivo correlations [100]. Three-dimensional tissue models have recently been demonstrated to produce better outcomes for drug screening than previous two-dimensional models in this pursuit, due to their capacity to imitate the spatial and chemical properties of actual tissues [100]. As a result, the industry can use these cost-effective, high-throughput, automated, and stable bioprinting procedures and equipment to generate human tissue models. To facilitate drug testing, more interactive disease models displaying important pathological characteristics should be bioprinted. Furthermore, the combination of 3D bioprinted tissue constructs with high-content readouts such as comprehensive genomic or proteomic expression analysis of biomarkers through bioinformatics data mining tools could well yield enormous volumes of complex data and open up an exciting future avenue for drug testing [101].

Furthermore, considering the major advances in engineering and healthcare that 3D bioprinting has enabled, bioprinting in four dimensions (4D) has become an area of increasing focus. 4D printing occurs when a printed 3D item is exposed to external energy inputs such as temperature, light, or other environmental stimuli to trigger a change [102]. 4D bioprinting can construct dynamic 3D patterned biological entities that change shape or behaviour in response to external inputs [103]. Multi-material prints with the potential to reshape over time, or a customized material system that can shift from one form to another, immediately off the print bed, are examples of 4D bioprinting [104]. This technique benefits from the development of smart materials, which can be designed to have a high degree of shape-changing potential. Recent efforts integrating naturally accessible polymers or hybrid smart materials have improved the ability to create volumetrically defined, cell-rich constructions with stimuli-responsive capabilities, shape memory properties, or dynamic motion in time [105]. For example, biocompatible stimuli-responsive shape memory hydrogels have been identified as interesting systems to use with this technology [106]. These materials are commonly used to assist cellular processes, as well as being able to be modified and mixed with other materials to obtain optimal properties for specific applications, making them extremely adaptable. Due to their inherent biocompatibility and biodegradability, intrinsic resemblance to natural tissues, ability to tune their properties through chemical modifications, and responsiveness to stimuli compatible with biological implementation, polymers of natural origin are being extensively investigated for “smart” bioink formulation. As a result, the 4D bioprinting approach has enabled the addition of several useful new ways to build engineered tissues. Vascularization, the capacity to execute a range of biological activities, and the integration of biophysical and biochemical signals to control cell fate and behaviour across time are all examples in the literature. These significant advancements make it straightforward to conclude that 4D bioprinting promotes enhanced integration with host tissues and functional regeneration [107]. Overall, 3D and 4D bioprinting strategies have the potential to revolutionize the field of tissue engineering.

## Figures and Tables

**Table 1:** Examples in literature of commonly used functionalized bioinks for tissue engineering applications.

Bioink Formulation	Application	Bioprinting Technique	Bioactivity	Advantages	Disadvantages	References
Bioprinted polyelectrolyte gelatin-CS (PGC) hydrogels with neonatal human foreskin fibroblasts (FBs)	Dermis constructs in which collagen and some blood vessels are produced	Extrusion	Cell viability was not quantified—images show high levels of viability	Good biocompatibility Good Attachment and proliferation	No functional assays were performed	[26]
Gelatin polymerized with thrombin loaded with human umbilical vein endothelial cells	Fully perfused vascularized 3D-bioprinted skin model	Extrusion and inkjet printing	Viability was not quantified, but the layers of skin were observed	Great printability Rapid gel–sol transition Recapitulation of skin phenotype and successful perfusion was achieved	The thrombin in the vascular bioink could partially crosslink the dECM–fibrinogen bioink during the incubation at 37 °C No information is provided on how long these constructs can be maintained in culture	[27]
Melanocytes (MCs) and Kupffer cells (KCs) on top of a bioprinted dermal layer consisting of a fibroblast-containing collagen hydrogel	Induced skin pigmentation upon subsequent air–liquid interface culture, creating a melanocytes-containing epidermal layer	Pneumatically driven extrusion-based printing	Cell viability was not quantified Histology showed the appropriate phenotypes were maintained	Good mechanical rigidity while having the printed cells kept in each layer at a designated depth	The use of immortalized KC could interfere with the proper differentiation of the KC (and incomplete stratification of the epidermis).	[28]
Gelatin–tyramine bioink encapsulated FBs (HDF and NIH-3T3) and HUVECs	Bioprinting endothelial cell-encapsulating gelatin–PEG–tyramine sheath was cultured in vitro and checked for blood vessel-like tissue formation	Coaxial-nozzle-based	High cell viability ranging from 80–95%	High biocompatibility and biodegradability Short gelling time Produced a relevant structure	Structure was only maintained in culture for eight days	[29]
Gelatin methacrylate and methacrylated alginate with neuroblastoma cells	Quantify and localize the effects of physical-chemical communication signals between tumour cells and the surrounding biomaterial stiffness over time	Extrusion	Cell proliferation (~30%) was observed	Measurements carried out in human tumours, mice tumours and hydrogels are comparable at room temperature	High elasticity in these hydrogels (Low Young’s Modulus)	[30]
